# Particularités du diabète de l’enfant et de l’adolescent au service d’endocrinologie du Centre Hospitalier Universitaire de Libreville de 2020 à 2023

**DOI:** 10.11604/pamj.2025.52.70.44443

**Published:** 2025-10-14

**Authors:** Nesta Patricia Ziza Ngaila, Daniela Nsame, Gladys Anguezomo, Treycia Pambo, Yasmine Ozavino Bakary, Pegguy Biloghe, Ludwine Bifoume Ndong, Philomène Kouna Ndouongo

**Affiliations:** 1 Département de Médecine et Spécialité Médicale, Service d'Endocrinologie, Centre Hospitalier Universitaire, Libreville, Gabon,; 2 Département de Médecine et Spécialité Médicale, Service de Neurologie, Centre Hospitalier Universitaire, Libreville, Gabon

**Keywords:** Enfants, adolescents, diabète de type 1, Children and adolescents, type 1 diabetes

## Abstract

**Introduction:**

le diabète de l'enfant et de l'adolescent constitue une préoccupation majeure en Afrique, du fait des difficultés de prise en charge et d'adhésion au traitement, avec comme risque la survenue précoce de complications dans cette population. L'objectif de cette étude était de faire un état des lieux de la population d'enfants et d'adolescents diabétiques suivis au Centre Hospitalier Universitaire de Libreville (CHUL).

**Méthodes:**

une étude transversale a été menée de janvier 2020 à décembre 2023 incluant les enfants et adolescents âgés de moins de 19 ans hospitalisés au service d'endocrinologie CHUL.

**Résultats:**

durant la période d'étude, 114 patients âgés de moins de 19 ans ont été hospitalisés, soit une fréquence hospitalière de 8%. La prédominance était féminine (sex ratio F/H 0,67). La moyenne d'âge était de 13,08 ± 4,14 ans, des extrêmes allant de 3 mois à 19 ans. L'âge moyen au diagnostic était de 10,96 ± 4,29 ans, 64,04% avaient une assurance maladie et 67,55% avaient un niveau secondaire. L'acidocétose représentait 64,91% des cas, la polyurie et la polydipsie étaient les signes les plus fréquents et 54,38% des patients avaient des auto-anticorps positifs. Les décompensations acido-cétosiques étaient fréquentes (29%). La mortalité représentait 3,5%.

**Conclusion:**

le diabète de l'enfant et de l'adolescent n'est pas une affection rare à Libreville. Il ressort qu'il touche plus les filles. Les complications les plus fréquentes sont la cétoacidose. La mortalité est faible; toutefois, le taux de réhospitalisations important pose le problème de l'éducation thérapeutique et de la transition dans nos populations de jeunes patients diabétiques.

## Abstract (en)

**Introduction:**

diabetes in children and adolescents is a major concern in Africa, due to challenges in management and treatment adherence, with the risk of early onset of complications in this population. The purpose of this study was to provide an overview of the population of diabetic children and adolescents followed at CHUL.

**Methods:**

we conducted a cross-sectional study from January 2020 to December 2023. It included children and adolescents under 19 years of age hospitalised in the Endocrinology Department of CHUL.

**Results:**

during the study period, 114 patients under the age of 19 were hospitalised, representing a hospital frequency of 8%. Female predominance was noted (F/M sex ratio 0.67). The mean age was 13.08 ± 4.14 years, with extremes ranging from 3 months to 19 years. The mean age at diagnosis was 10.96 ± 4.29 years, 64.04% had health insurance, and 67.55% had a secondary education level. Diabetic ketoacidosis accounted for 64.91% of cases; polyuria and polydipsia were the most frequent signs, and 54.38% of patients tested positive for autoantibodies. Ketoacidosis decompensations were frequent (29%). Mortality accounted for 3.5% of cases.

**Conclusion:**

diabetes in children and adolescents is not a rare condition in Libreville. It appears to affect girls more frequently. The most common complication is ketoacidosis. Mortality is low; however, the high rate of rehospitalisations raises the issue of therapeutic education and transition in our population of young diabetic patients.

## Introduction

Le diabète est un groupe de maladies métaboliques liées à une hyperglycémie chronique résultant d'un défaut de la sécrétion de l'insuline ou de l'action de l'insuline ou de ces deux anomalies associées [1]. Il fait partie de la liste des 10 principales maladies non transmissibles et de décès dans le monde. Il touche aussi bien les enfants, les sujets jeunes et les adultes. La prévalence mondiale du diabète était de 537 millions en 2021 avec 24 millions en provenance du continent africain [2]. Ce nombre ne cesse d'augmenter et l'Afrique connaîtra une forte incidence s'estimant à 55 millions de cas d'ici à 2045 [2]. Parallèlement, l'Afrique subsaharienne, est confrontée au double fardeau lié aux maladies non transmissibles et transmissibles. Les enfants et les adolescents n'en sont pas épargnés. En effet, la prévalence mondiale du diabète de type 1 était d'environ 542 000 sur une population de 1,9 million d'enfants du même âge [3]. Le diabète de type 1 est la forme la plus courante dans cette tranche d'âge [3]. Il correspond à plus de 90% des diabètes de l'enfant et de l'adolescent. Entre 2011 et 2021, l'Afrique a enregistré une multiplication par cinq du nombre de cas de diabète de type 1 chez les enfants et les adolescents de moins de 19 ans, passant de quatre pour 1000 enfants à près de 20 pour 1000 [4].

Au Gabon, la prévalence du diabète a été estimée par la FID à 6% en 2021 [2]. Le centre spécialisé dans la prise en charge des patients diabétiques est le service d'endocrinologie du Centre Hospitalier Universitaire de Libreville (CHUL). En effet, au sein de ce service, le diabète est le premier motif d'hospitalisation et les enfants représentent une proportion non négligeable des admissions. Ntyonga *et al*. dans une étude réalisée dans ce même service environ 25 ans auparavant retrouvaient que le diabète chez l'enfant était rare, la forme prédominante était le diabète de type 1 avec un âge à la découverte entre 7 et 15 ans et l'acidocétose diabétique [5]. En 2019, Damiens *et al*. [6] ont également mené une étude qui retrouvait plus d'enfants et d'adolescents diabétiques, devant les modifications de caractère des populations, le polymorphisme du diabète et l'amélioration des conditions de prise en charge. Nous avons voulu actualiser les informations relatives au suivi des enfants diabétiques des quatre dernières années. L'objectif de notre étude était de faire un état des lieux du diabète dans cette population au service d'Endocrinologie du Centre Hospitalier Universitaire de Libreville (CHUL) de 2020 à 2023.

## Méthodes

**Conception de l'étude:** une étude rétrospective à visée descriptive a été menée pour établir un état des lieux du suivi des patients diabétiques de type 1 à Libreville

**Cadre de l'étude et population:** cette étude a été menée au CHUL, qui est l'un des plus grands hôpitaux du pays, situé au centre de Libreville, capitale politique du Gabon. Il s'agit d'un centre hospitalier universitaire polyvalent, qui propose quasiment toutes les spécialités médicales. Parmi les 10 services de médecine au sein du CHUL, le service d'endocrinologie et des maladies métaboliques représente le centre de référence de la prise en charge du diabète et des maladies métaboliques.

**Population d'étude:** elle était composée de dossiers médicaux de tous les patients diabétiques âgés de moins de 19 ans hospitalisés au service d'endocrinologie entre 2020 et 2023 comportant les données nécessaires.

**Variables:** elles comprennent les caractéristiques sociodémographiques de la population d'étude, les caractéristiques du diabète (date de début du diabète, mode de révélation, mode de décompensation, antécédents familiaux de diabète, traitement du diabète, les signes cliniques et biologiques) et l'évolution de la maladie. Le bilan biologique comprenait le dosage de l'hémoglobine glyquée (HbA1C), la glycémie veineuse et le dosage des autoanticorps.

**Outil de collecte de données:** les données relatives à tous les aspects de notre étude ont été collectées à l'aide d'un questionnaire rédigé en français, conçu spécifiquement pour notre recherche. Ce questionnaire a ensuite été intégré dans l'application mobile KoboCollect, outil utilisé pour la collecte.

**Analyse de données:** la complétude des données recueillies ainsi que leur cohérence ont été vérifiées quotidiennement. Les données ont ensuite été saisies et l'analyse des données s'est faite avec le logiciel Epi Info 7.2.5.0. Nous avons procédé à la description de la distribution de la population d'étude. Les variables quantitatives ayant une distribution normale ont été présentées selon leur moyenne et leur écart-type. Les variables ayant une distribution asymétrique ont été présentées avec leur médiane et leur intervalle interquartile (IIQ). Les proportions ou pourcentages ont été calculés pour les variables qualitatives. Les données ont été considérées statistiquement significatives pour une valeur de P inférieure à 0,05.

**Considérations éthiques:** des autorisations ont été accordées par la Direction Générale du CHUL et le chef du service d'endocrinologie pour l'accès aux archives dudit service. Aucune information personnelle identifiable n'a été collectée et la confidentialité a été assurée. Les données électroniques ont été stockées dans un ordinateur protégé par un mot de passe.

## Résultats

**Caractéristiques sociodémographiques:** durant la période d'étude, 114 patients diabétiques âgés de moins de 19 ans ont été hospitalisés avec une fréquence hospitalière estimée à 8%. La population d'étude était à prédominance féminine avec un sex-ratio F/H de 0,67. L'âge moyen était de 13,08 ± 4,14 ans, avec des extrêmes allant de 03 mois et 19 ans et l'âge moyen au diagnostic était de 10, 96 ± 4,29 ans. Le Tableau 1 résume les données sur les caractéristiques sociodémographiques de la population

**Tableau 1:** caractéristiques sociodémographiques des patients

Caractéristiques sociodémographiques	Effectif	%
**Sexe**		
Féminin	68	59,65
Masculin	46	40,35
**Age**		
< 5	6	6,14
5-10	16	14,04
10-15	45	39,47
>15	47	41,23
**Niveau d'instruction**		
Non scolarisé	6	5,26
Primaire	29	25,44
Secondaire	77	67,55
Universitaire	2	1,75
**Assurance maladie**		
Oui	73	64,04
Non	41	35,96
**Provenance**		
Consultations externes	43	37,72
Urgences pédiatriques	36	31,58
Domicile	29	25,44
Réanimation	6	5,26
**Histoire de la maladie**		
Diabète Novo	60	52,63
Diabète Ancien	54	47,37

**Figure 1:**
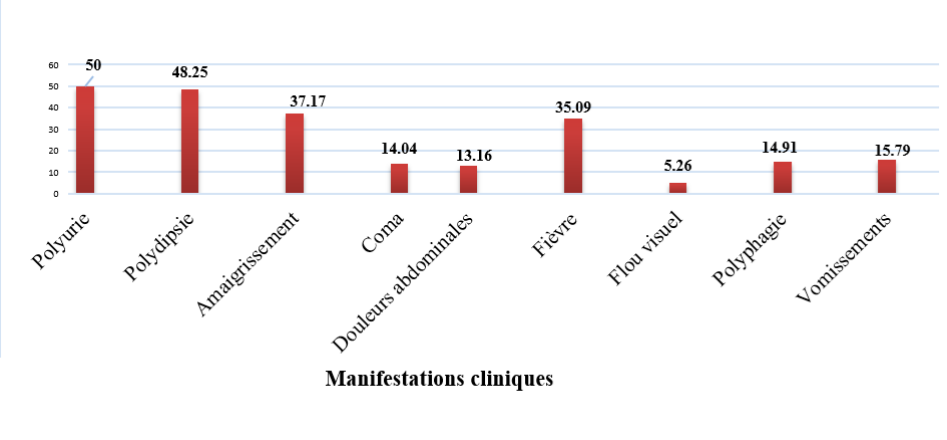
caractéristiques cliniques des patients

**Analyse descriptive:** parmi les 114 enfants et adolescents diabétiques, 26,31% avaient un antécédent familial de diabète. Les circonstances de découverte ont été l'acido-cétose dans 60% des cas. Le diabète de Novo représentait 52,33%. Les patients diabétiques connus avaient une durée moyenne d'évolution de la maladie de 3,76 ± 3,42 ans. Les manifestations cliniques du diabète sont résumées dans la Figure 1. L'acidocétose représentait 64,91% des cas et l'hyperglycémie sans cétose 35,09%. Concernant le traitement 99,22% des patients étaient sous insulinothérapie, dont 89,47% sous le protocole basal-bolus. La durée moyenne d'hospitalisation était de 6,92 jours ± 3,09. L'évolution était marquée par les décompensations acido-cétosiques fréquentes chez 42 patients, soit 29%, qui avaient été plusieurs fois réhospitalisés durant les trois années de l'étude pour acido-cétose. La mortalité représentait 3,5% de la population d'étude. Le Tableau 2 résume les données biologiques des patients.

**Tableau 2:** caractéristiques biologiques des patients

Variables	Effectif	Pourcentage %
**Acétonurie**		
Négative	22	19,30
Positive	92	80,70
**Glucosurie**		
Négative	24	21,05
Positive	90	78,95
**Glycémie (mg/dl)**		
<80	1	1,09
80-250	66	71,74
>600	25	27,17
**HbA1c (%)**		
<6	1	2,27
6-7	0	00
7-10	13	29,55
>10	30	68,18
**Auto anticorps**		
Positif	62	54,38
Négatif	52	45,61

## Discussion

Le diabète chez l'enfant au Gabon occupe une place préoccupante. Sa fréquence hospitalière dans la présente étude était de 8%. Elle était largement au-dessus de celle retrouvée par Monabeka *et al*. au Congo (2,8%) [7]. Issoufou *et al*. au Mali retrouvaient cependant une fréquence hospitalière plus élevée de 34,32% [8]. Cette différence pourrait être la conséquence de la taille faible de l'échantillon par rapport à celle des autres séries.

La répartition du sexe selon les différentes études reste hétérogène. Le sexe féminin prédominait et ce même constat était fait par Lokrou *et al*. en Côte d'Ivoire [9] et Issoufou *et al*. au Mali [8]. Une prédominance masculine était toutefois retrouvée par Liman El-Hadji *et al*. au Mali avec 13 garçons (65%) et 7 filles (35%) [10]. Certains auteurs ont pu démontrer qu'il n'existait pas de différence entre l'incidence du diabète chez les filles et les garçons [11]. Comme dans la plupart des études, le diabète chez l'enfant est plus fréquent au-delà de 10 ans. Dans ce travail, l'âge moyen de la population était estimé à environ 13,08 ans, similaire à l'âge moyen retrouvé par Togo *et al*. au Mali (13ans) [12] Concernant le pic de fréquence de la maladie il se situait entre 15 et 19 ans, Monabeka *et al*. retrouvaient que la tranche d'âge la plus touchée par le diabète était celle de 14 à 19 ans [7].

La majorité des patients était scolarisée (94,73%), Valentine *et al*. au Cameroun constataient également dans leurs travaux que plus de la moitié des patients étaient scolarisés [13]. Le niveau d'instruction prédominant était le niveau secondaire (67,55%), ce constat pourrait s'expliquer par les tranches d'âge prédominantes correspondant à ce niveau de scolarisation, mais aussi par le taux de scolarisation au Gabon qui était en 2021 supérieur à 94% [14]. Un antécédent familial de diabète était retrouvé chez 26,31% des patients. Togo *et al*. au Togo et Valentine *et al*. au Cameroun rencontraient respectivement 31,4% et 50% de patients ayant un antécédent familial [12,13,15]. Cela peut se concevoir par le poids de l'hérédité qui est plus ou moins important selon le type de diabète. En effet, chez une personne ayant un diabète de type 1, le risque est estimé à 8% si le père est diabétique, 4% si c'est le cas de la mère et 30% si les deux sont atteints. Le mode de révélation cétosique était rencontré chez 81,48% des patients et la survenue brutale du diabète chez l'enfant et l'adolescent était rapportée par tous les auteurs. Ce qui résulte du statut méconnu de la maladie malgré une évolution insidieuse depuis quelques semaines, passée inaperçue, mais également du caractère insulinopénique traduisant la destruction brutale des cellules bêta des îlots de Langerhans au cours du diabète de type 1. L'acidocétose, révélatrice de la maladie peut être une situation grave pourvoyeuse de morbidités et de mortalité.

Parmi les patients qui avaient réalisé l'HbA1c, 97,67% avaient une hémoglobine glyquée supérieure à 7%, au Mali, 79,7% des patients avaient une HbA1c supérieure à 7%. Ces valeurs pourraient s'expliquer par le statut de diabète nouvellement diagnostiqué évoluant déjà depuis quelques semaines en ce qui concerne le diabète de novo et pour les patients déjà suivis, l'équilibre du diabète cible dans cette population de patients jeunes est le plus souvent difficile du fait de la transition et des différents stades d'acceptation de la maladie. Presque la totalité des patients était sous insulinothérapie. Ce constat était fait au Mali où 98,4% des patients utilisaient l'insuline et majoritairement le protocole basal-bolus. Ceci peut s'expliquer par les caractéristiques épidémiologiques de l'échantillon fortement constitué de diabétiques de type 1 chez qui le traitement insulinique vise à reproduire la sécrétion physiologique insulinique par les injections préprandiales et une injection d'insuline basale au coucher. Toutefois, on a noté que certains patients étaient sous insuline prémixée à deux injections. Ce protocole de pis-aller est le plus souvent administré chez des patients démunis ne pouvant s'octroyer l'insuline, mais plus fréquemment chez des patients ne pouvant avoir les trois repas rythmés par le nombre d'injections dans le basal-bolus.

Le décès dans la présente étude représentait 3,5% des cas. Cette donnée est inférieure aux sept cas de décès retrouvés par Sarr *et al*. [16]. Cette faible mortalité constatée peut être liée à la bonne accessibilité aux soins des enfants diabétiques et à la couverture nationale estimée à plus de 60% de l'assurance maladie qui réduit considérablement le coût de la prise en charge [17]. A cela peuvent s'ajouter la standardisation et l'efficacité des protocoles de prise en charge de l'acido-cétose diabétique dans les services d'endocrinologie du CHUL, la formation du personnel soignant et l'éducation thérapeutique des enfants et leurs familles.

## Conclusion

Le diabète de l'enfant et de l'adolescent n'est pas une affection rare à Libreville, sa fréquence dans notre étude était de 8%. Les principales manifestations cliniques observées au début de la maladie étaient les signes cardinaux et la principale complication, la cétoacidose diabétique. L'âge moyen au moment du diagnostic était d'environ 10 ans et la majorité des patients avaient une durée d'évolution de la maladie de moins de quatre ans. La prise en charge standardisé et précoce de nos patients justifie le faible taux de mortalité dans notre étude, toute fois le taux de réhospitalisassions important retrouvé pose le problème de l'éducation thérapeutique et de la transition dans nos populations de jeune diabétique.

### Etat des connaissances sur le sujet

- Le diabète de l'enfant et de l'adolescent reste prédominé par le diabète de type 1 qui est moins fréquent en Afrique comparé aux pays occidentaux, mais cette faible prévalence est probablement liée à un sous-diagnostic;
- Les inégalités d'accès aux soins, les superstitions ou croyances culturelles et le manque d'éducation de la population peuvent retarder le diagnostic et la prise en charge de ces enfants; le décès survient très souvent avant même le diagnostic ou peu après le diagnostic;
- La période critique de la transition dans le diabète de type 1 entraîne de nombreuses difficultés, impactant la santé, l'adhérence au traitement et la qualité de vie du jeune adulte.

### Contribution de notre étude à la connaissance

- Malgré la prévalence élevée du diabète chez l'enfant et l'adolescent dans le monde, il existe très peu d'informations concernant l'épidémiologie du diabète de l'enfant et de l'adolescent au Gabon;
- Cette étude montre que le diabète de l'enfant et de l'adolescent n'est pas une affection rare à Libreville; la disparité des profils cliniques rend parfois difficile la caractérisation du diabète chez l'enfant bien que dans la plupart des cas il s'agit d'un diabète de type 1 auto-immun;
- L'assurance maladie nationale rend accessible la prise en charge des enfants et adolescents, mais l'éducation thérapeutique, très peu développée dans notre contexte, serait fondamentale pour, éviter les complications, les réhospitalisations, et favoriser l'autonomie progressive des enfants et de leurs aidants.
